# Heat Stress Causes Immune Abnormalities via Massive Damage to Effect Proliferation and Differentiation of Lymphocytes in Broiler Chickens

**DOI:** 10.3389/fvets.2020.00046

**Published:** 2020-02-07

**Authors:** Ryota Hirakawa, Siti Nurjanah, Kyohei Furukawa, Atsushi Murai, Motoi Kikusato, Tomonori Nochi, Masaaki Toyomizu

**Affiliations:** ^1^Laboratory of Animal Nutrition, Division of Life Sciences, Graduate School of Agricultural Science, Tohoku University, Sendai, Japan; ^2^International Education and Research Center for Food and Agricultural Immunology, Graduate School of Agricultural Science, Tohoku University, Sendai, Japan; ^3^Laboratory of Animal Nutrition, Department of Animal Sciences, Graduate School of Bioagricultural Sciences, Nagoya University, Nagoya, Japan; ^4^Laboratory of Functional Morphology, Division of Life Sciences, Graduate School of Agricultural Science, Tohoku University, Sendai, Japan; ^5^International Research and Development Center for Mucosal Vaccine, Institute of Medical Science, The University of Tokyo, Tokyo, Japan

**Keywords:** heat stress, broiler chickens, immunity, bursa of fabricius, thymus, spleen

## Abstract

Broiler chickens are highly sensitive to high ambient temperatures due to their feathers, lack of skin sweat glands, and high productivity. Heat stress (HS) is a major concern for the poultry industry because it negatively affects growth as well as immune functions, which increase the potential risk of infectious disease outbreaks. Therefore, it is vital to elucidate HS's effect on the avian immune system, especially considering the global rise in average surface temperature. Our study identified a series of immunological disorders in heat-stressed broiler chickens. We exposed 22-day-old broiler chickens to a continuous HS condition (34.5 ± 0.5°C) for 14 days and immunized them with a prototype bovine serum albumin (BSA) antigen. The plasma and lymphoid tissues (thymus, bursa of Fabricius, and spleen) were harvested at the end of the experiments to investigate the induction of BSA-specific immune responses. Our results revealed that plasma titers of immunoglobulin (Ig)Y, IgM, and IgA antibodies specific for BSA were lower than those of thermoneutral chickens immunized with BSA. Furthermore, the spleens of the heat-stressed broiler chickens displayed severe depression of Bu1^+^ B cells and CD3^+^ T cells, including CD4^+^ T cells and CD8^+^ T cells, and lacked a fully developed germinal center (GC), which is crucial for B cell proliferation. These immunological abnormalities might be associated with severe depression of CD4^−^CD8^−^ or CD4^+^CD8^+^ cells, which are precursors of either helper or killer T cells in the thymus and Bu1^+^ B cells in the bursa of Fabricius. Importantly, HS severely damaged the morphology of the thymic cortex and bursal follicles, where functional maturation of T and B cells occur. These results indicate that HS causes multiple immune abnormalities in broiler chickens by impairing the developmental process and functional maturation of T and B cells in both primary and secondary lymphoid tissues.

## Introduction

A high ambient temperature is one of most severe environmental stressors for poultry. It is associated with performance degradation, inefficient feed use, and even increased mortality (1–3). Unlike mammals, chickens dissipate excess heat by panting to prevent body temperature elevation, but they often experience disorders of systemic energy homeostasis due to poor feed intake under the heat stress (HS) condition (4, 5). Along with performance degradation, under the HS condition, the weights of major organs (e.g., pectoral muscle and liver) do not increase as expected (6, 7). Additionally, the weight of lymphoid tissues (thymus, bursa of Fabricius, and spleen) is also reduced (3, 8–10). However, few studies that demonstrate the structural and functional changes of such lymphoid tissues have been conducted.

Vaccination is an effective strategy for inducing adaptive immune responses for preventing the onset of infectious diseases caused by pathogenic bacteria or viruses. This common immunotherapy has been utilized for industrial animals including chickens (11). In the poultry industry, several vaccines have been used, especially for controlling infectious bronchitis, and Newcastle disease. However, under the HS condition, the titer of antibodies that react to pathogens does not increase as expected by such vaccinations in broiler chickens (9, 12), suggesting that HS deeply influences their immune function. Another important finding related to weak immune reactions in heat-stressed broiler chickens is the increased proportion of pathogenic bacteria, such as *Salmonella* sp., *Clostridium* sp., and *Escherichia coli*, in the gut (13–15). Increased enteric permeability in the gut of heat-stressed broiler chickens might also be associated with disorders of intestinal immune functions (15–18) and the promotion of bacterial translocation from the gut to other organs (17, 19, 20). In fact, the frequency of gut-derived *Salmonella* sp. in the liver and muscle increases under the HS condition (21). Therefore, future poultry science research focusing on avian immunology must be designed to advocate an appropriate approach healthy chicken production, even under the HS condition.

The potential risk of disease outbreaks in commercial poultry is increasing due to high stocking densities and high yield requirements of the rapidly developing poultry industry because of the increased global demand for poultry meat (22–24). In addition, we should not forget that the poultry industry has a risk of spreading avian influenza virus subtypes H9N2, H5N1, H5N8, and H7N9, which recently spread worldwide, originated from the wild birds (25–28). Furthermore, increasing warmer temperatures can alter the timing and patterns of bird migration, creating novel assemblages of species, and new opportunities for viral transport and reassortment (29). Previous studies have demonstrated that HS decreases immunocompetence in chickens, such as decreased the weights of immune tissues (3, 8–10), decreased antibody production against antigens immunized (9, 12), alteration of the expression level of inflammatory cytokines of the spleen and cecal tonsil (10, 30, 31), and decreased macrophage activity (3). Although the number of heat-stressed broiler chickens has been obviously increasing with global warming, it has not been still enough to elucidate the molecular and cellular mechanisms underlying the immunosuppression observed in heat-stressed broiler chickens. Therefore, the alteration of tissue structure constructed by immune cells in lymphoid tissues under the HS condition must be investigated in detail by histological and immunological analyses to create a new breeding strategy that adapts to heat-stressed broiler chickens to minimize the burden of infectious diseases and increase productivity.

In this study, we examined the status of immune development in heat-stressed broiler chickens compared to chickens reared under the thermoneutral (TN) condition. We also determined the alteration of immune functions under the HS condition by immunization with a prototype antigen. Our results clearly demonstrate that HS causes severe damage, especially to the developmental process and functional maturation of the immune system in primary and secondary lymphoid tissues.

## Materials and Methods

### Animals

All animal experiments were conducted in accordance with the principles of the Basel Declaration and approved by the Tohoku University Institutional Animal Care and Use Committee. A total of 60–80 chicks (Ross strain, *Gallus gallus domesticus*, male, 0 days old), which were hatched in a commercial hatchery (Miyagi, Japan), were purchased and used for each experiment. All chicks were housed in electrically-heated battery cages with 24-h light until being used in our experiment. At 15 days old, the chicks were moved into individual wire cages that were maintained at 24 ± 0.5°C until they were 22 days old so they could adapt to their surroundings. At 22 days old, the chickens were weighed to confirm normal growth and divided into a thermoneutral (TN) and HS groups. In the HS group, the temperature was increased gradually by 1°C per hour until reaching 34.5 ± 0.5°C, and was maintained until all experiments were completed. The daylight hours remained unchanged. The humidity of the animal facility was adjusted to 55% or less throughout the experiments. The chickens had *ad libitum* access to a corn-soybean basal diet (22% of crude protein and 3,100 kcal/kg of metabolizable energy) and water.

### Immunization

Three independent immunization studies were conducted. When the chickens were 25 days old, we immunized 16 (trial 1), 17 (trial 2), and 24 (trial 3) of them intramuscularly (in the left breast muscle) with 10 μg of bovine serum albumin (BSA; Nacalai Tesque Inc., Kyoto, Japan) dissolved in 200 μl of sterilized phosphate-buffered saline (PBS) and boosted the immunization with the same dose of BSA seven days later (at 32 days old). Of the 16, 17, and 24 chickens, we exposed 10, 11, and 12 to the HS condition (34.5 ± 0.5°C) for 14 days (3 days before initial immunization and 4 days after booster immunization), and the remainder were kept in the TN condition (24 ± 0.5°C). As a control, another 14 (trial 1), 15 (trial 2) 22 (trial 3) chickens were divided into TN (*n* = 6, 6, 10) and HS (*n* = 8, 9, 12) groups and administered 200 μl of sterilized PBS only via intramuscular injection. The performance parameters of control chickens were precisely characterized.

### Body Weight and Cumulative Feed Intake

To confirm the influence of ambient temperature on animal performance and stress responses, chickens in the TN (*n* = 12) and HS (*n* = 12) groups were monitored for changes in animal body weight (ABW) and cumulative feed intake during the experiment (22–36 days old). Three independent studies were conducted.

### Flow Cytometry

We harvested a piece of lymphoid tissues from the thymus, bursa of Fabricius, and spleen of 36-day-old chickens (TN, *n* = 8; HS, *n* = 8) and dispersed mechanically on the Falcon™ Cell Strainers (Life Sciences) to isolate cells after measuring the weight of the piece and whole tissue. The number of isolated cells present in the piece of tissue was manually counted under a microscope and the total number of cells present in whole tissue was calculated based on the weight measured. The cells were stained for 30 min at 4°C with fluorescein isothiocyanate (FITC)—conjugated mouse anti-chicken CD3 (clone CT-3; 500 ng/ml; Southern Biotech, Birmingham, AL, USA), phycoerythrin (PE)-conjugated mouse anti-chicken Bu1 (clone AV20; 100 ng/ml; Southern Biotech), and allophycocyanin-conjugated mouse anti-chicken CD45 (clone LT40; 100 ng/ml; Southern Biotech). To investigate T cell subsets, the cells were stained with FITC-conjugated mouse anti-chicken CD3 (clone CT-3), PE-conjugated mouse anti-chicken CD4 (clone EP96; 100 ng/ml; Southern Biotech), and Cy5-conjugated mouse anti-chicken CD8α (clone CT-8; 100 ng/ml; Southern Biotech). Via-Probe (10 μl/stain; BD Biosciences) was added to the stains to exclude dead cells from the analyses. The cells were finally analyzed using a flow cytometer Accuri C6 (BD Biosciences, Franklin Lakes, NJ) and the absolute number of T, B, and CD4^+^ and CD8^+^ cells was determined based on the frequency of the cells and the total number of cells present in the tissue according to the previous studies (32, 33).

### Histochemistry

Lymphoid tissue samples (thymus, bursa of Fabricius, and spleen) harvested from 36-day-old chickens (TN, *n* = 8; HS, *n* = 8) were immediately fixed in 4% (w/v) paraformaldehyde for 24 h. Following dehydration of 12 h each with 70, 80, 90, 95, 100, and 100% (v/v) ethanol, the tissues were embedded in paraffin, and 5-μm thick sections were stained with hematoxylin and eosin. We observed the morphological characteristics of the thymus, bursa of Fabricius and spleen samples via light microscopy (AX70; Olympus, Tokyo, Japan).

### Plasma Collection

We collected blood samples from all chickens' wing veins before immunization (at 25 days old), 4 days after initial immunization (at 29 days old), and following booster immunization (at 36 days old). The plasma samples were separated from the whole blood by centrifugation at 1,500 × *g* for 10 min at 4°C and stored at −20°C for use in enzyme-linked immunosorbent assays (ELISAs).

### Enzyme-Linked Immunosorbent Assays

The plasma corticosterone (CORT) concentration was measured at 36 days old using a commercially available ELISA kit (ADI-900-097; Enzo Life Sciences Inc., Farmingdale, NY) according to the manufacturer's instructions and previous studies (34, 35). We also determined the concentrations of total immunoglobulin (Ig) subclasses IgY, IgM, and IgA and BSA-specific titers by ELISA. For the Ig subclasses, 96-well ELISA plates (Thermo Fisher Scientific, Waltham, MA) were coated overnight at 4°C with 500 ng/ml of goat anti-chicken IgY, IgM, or IgA polyclonal antibodies (Bethyl Laboratories, Montgomery, TX, USA) and then blocked for 1 h at room temperature (RT) with 1% (w/v) of BSA and 0.05% (v/v) of Tween-20. To investigate BSA-specific antibody titers, other plates were coated overnight at 4°C with 1 μg/ml of BSA and then blocked for 1 h at RT with 0.05% (v/v) of Tween-20. Serially diluted plasma samples were separately added to each well of both plates and incubated for 2 h at RT. Chicken reference serum in which chicken IgY, IgM, and IgA with known concentrations were included was used as standard for total Ig analysis. A commercial indirect ELISA sets were used to quantify total IgY, IgM, and IgA concentrations according to the manufacturer's instructions (Bethyl Laboratories, Montgomery, TX, USA) in chicken reference serum. After washing, the plates were treated with 100 ng/ml of Ig subclass-specific goat polyclonal antibodies conjugated with horseradish peroxidase (Bethyl Laboratories, Montgomery, TX, USA) as secondary antibodies for 1 h at RT. The signals were developed using a tetramethylbenzidine microwell peroxidase substrate system (KPL, SeraCare Life Sciences, Milford, MA).

### Statistics

Statistical significance was tested by one-way analysis of variance followed by Tukey's test using R 3.4.2 for Mac OS X Cocoa GUI or an unpaired two-tailed Student's *t*-test using Excel 2016 software. Data were presented as mean ± standard error (SE). Pearson correlation coefficients were estimated to quantify the relationships between two parameters using R 3.4.2 for Mac OS X Cocoa GUI. Differences were considered significant for *p* < 0.05 in all statistical analyses.

## Results

### Lymphoid Tissues Are Particularly Sensitive Organs to HS in Broiler Chickens

**Figure 1:**
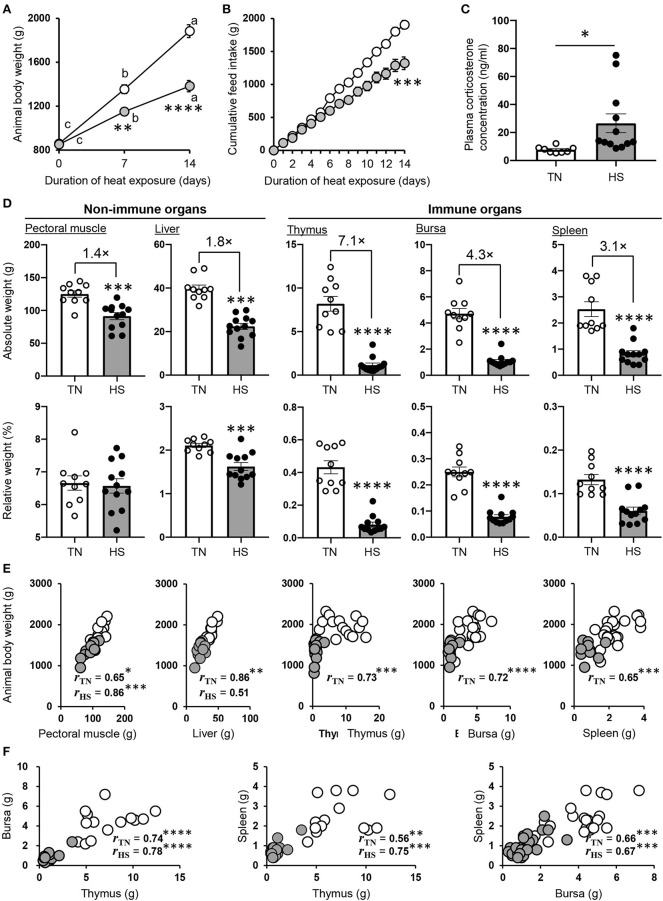
Performance parameters of broiler chickens under the HS condition. **(A,B)** The chickens were maintained under the TN (24 ± 0.5°C) or HS (34.5 ± 0.5°C) condition. The HS and TN conditions are indicated by gray and clear circles, respectively. Animal body weight **(A)** and cumulative feed intake **(B)** of each group were measured for 2 weeks (22–36 days old). **(C)** The plasma CORT concentration was analyzed in both groups at 36 days of age. **(D)** The pectoral muscle, liver, thymus, bursa of Fabricius, and spleen were harvested from each group at 36 days of age, and their absolute and relative weights were measured. Three experiments were conducted and a representative result was shown in **(A–D)**. **(E,F)** The relationship between body weight, the weight of each tissue **(E)**, and among the weight of lymphoid tissues **(F)** obtained from all three experiments conducted were evaluated by Pearson correlation analyses. The data are presented as means ± SE (*n* = 10–12/group for animal body weight and cumulative feed intake, *n* = 10–12/group for tissue weight and plasma CORT concentration). *p < 0.05, **p < 0.01, ***p < 0.001, ****p < 0.0001 compared with the TN group (unpaired two-tailed Student's *t-*test). **(A)** For points a, b, and c, *p* < 0.05 compared among each group for the different time points.

Consistent with the previous studies (1, 2), ABW and cumulative feed intake were both progressively elevated throughout the experimental period regardless of the breeding condition; however, these parameters were much higher in the TN group than the HS group (Figures 1A,B). The concentration of plasma CORT, which is a stress marker, was higher in the HS group than the TN group (Figure 1C). A poor ABW gain in the HS group was observed in all organs investigated (pectoral muscle, liver, thymus, bursa of Fabricius, and spleen). Of note, there was a substantial difference between the TN and HS groups in the weight of immune organs, including both primary, and secondary lymphoid tissues (Figure 1D).

We wondered whether the poor growth performance caused by HS was due to its influence on lymphoid tissue development. Thus, we next calculated the percentage of each tissues to ABW (relative weight). Although there was no or slight difference in relative weight of non-immune tissue among experimental groups, that of immune organs was extremely low in the HS group compared to the TN group (Figure 1D). These results indicated that the atrophy of lymphoid tissues might be not associated with only poor growth performance but a series of HS-induced disorders (e.g., malnutrition, inflammation, oxidative stress). To explore the correlation between ABW and the weight of both lymphoid and non-lymphoid tissues in detail, we conducted Pearson correlation analyses with corresponding data sets obtained from three animal trials. Correlations between ABW and the weight of pectoral muscle, liver, thymus, bursa of Fabricius, and spleen are presented in Figure 1E. Significant correlations were observed between ABW and the weight of non-immune tissues, namely, pectoral muscle (rTN = 0.65, *p* = 0.041; rHS = 0.86, *p* = 0.0003) and liver (rTN = 0.86, *p* = 0.0014; rHS = 0.51, *p* = 0.087), in both TN and HS conditions. Importantly, a much stronger correlation was observed in the TN condition between ABW and the weight of immune tissues, namely, thymus (rTN = 0.73, *p* = 0.0001), bursa of Fabricius (rTN = 0.72, *p* = 0.000016), and spleen (rTN = 0.65, *p* = 0.0001); however, these correlations were not seen at all in the HS condition. Among the lymphoid tissues, significant and strong correlations were observed in the weight when each organ was compared to the counterpart organ of the individual chicken reared in both the TN and HS groups (Figure 1F). These results indicate that the HS-induced reduction of growth performance could decrease the weight of pectoral muscle and liver due insufficient basal metabolism but may not immediately cause severe shrinkage of lymphoid tissues.

### Antigen-Specific Humoral Immune Responses Are Depressed by HS

**Figure 2:**
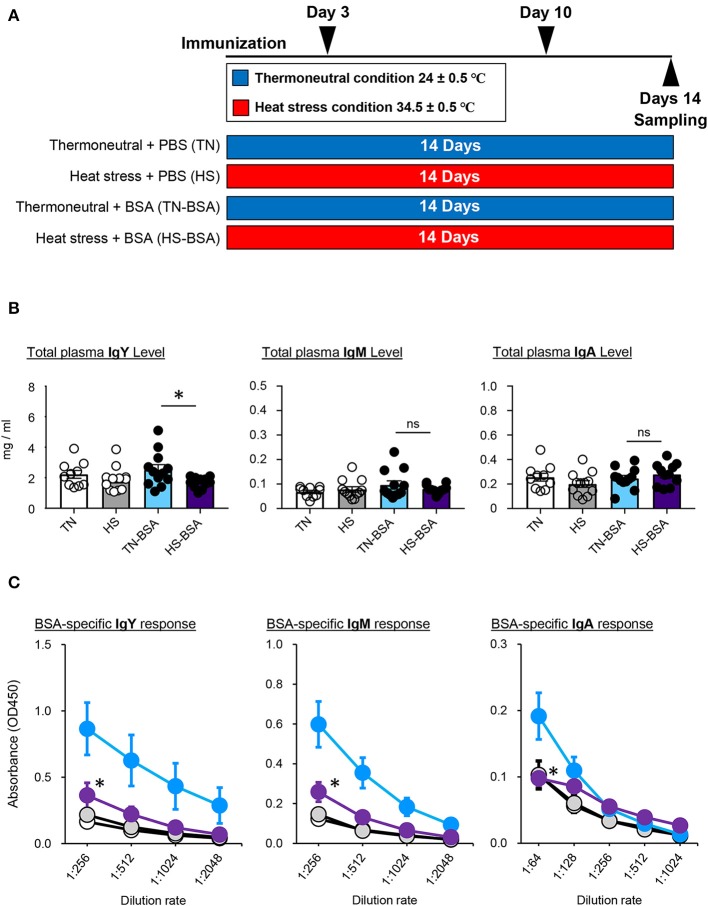
Antigen-specific immune response of broiler chickens reared in the HS condition **(A)**. The experimental schedule is summarized. All birds were maintained in either TN or HS condition throughout the 14-days experimental period. To assess antigen-specific immune responses of heat-stressed chickens, broiler chickens under the TN (TN-BSA) and HS (HS-BSA) conditions were immunized intramuscularly with 10 μg of BSA on days 3 and 10 of the experiment. As a control, other broiler chickens, maintained under the TN and HS conditions, were immunized intramuscularly with phosphate-buffered saline on days 3 and 10 of the experiment. Blood samples were collected from the wing veins of all chickens 4 days after initial and booster immunization **(B,C)**. Total plasma IgY, IgM, and IgA levels **(B)** and BSA-specific IgY, IgM, and IgA responses **(C)** were analyzed by ELISA. Three experiments were conducted and a representative result was shown. The data are presented as means ± SE (*n* = 10–12/group). *p < 0.05 compared between TN-BSA and HS-BSA (unpaired two-tailed Student's *t*-test). Clear, TN; gray, HS; blue; TN-BSA; purple, HS-BSA.

To investigate the immune function under the HS condition, we next immunized broiler chickens from the TN and HS groups intramuscularly with a prototype BSA antigen (Figure 2A). The total amounts of IgY, IgM, and IgA antibodies in plasma were not significantly elevated by immunization regardless of the presence or absence of HS; however, the amount of total IgY following immunization was lower in the HS group than the TN group (Figure 2B). Next, we addressed the effect of HS on the induction of antigen-specific antibody production. The titers of BSA-specific IgY and IgM antibodies were elevated after immunization in both the TN and HS conditions (Figure 2C). Nevertheless, the titers of BSA-specific IgY and IgM antibodies were much lower in the HS group compared to the TN group (Figure 2C). BSA-specific IgA antibodies were detected after immunization in the TN group only (Figure 2C). These results indicate that immunization under the HS condition influence the induction of antigen-specific antibody production in broiler chickens.

### HS Induces a Substantial Difference in the Number of Splenic Lymphocytes

**Figure 3:**
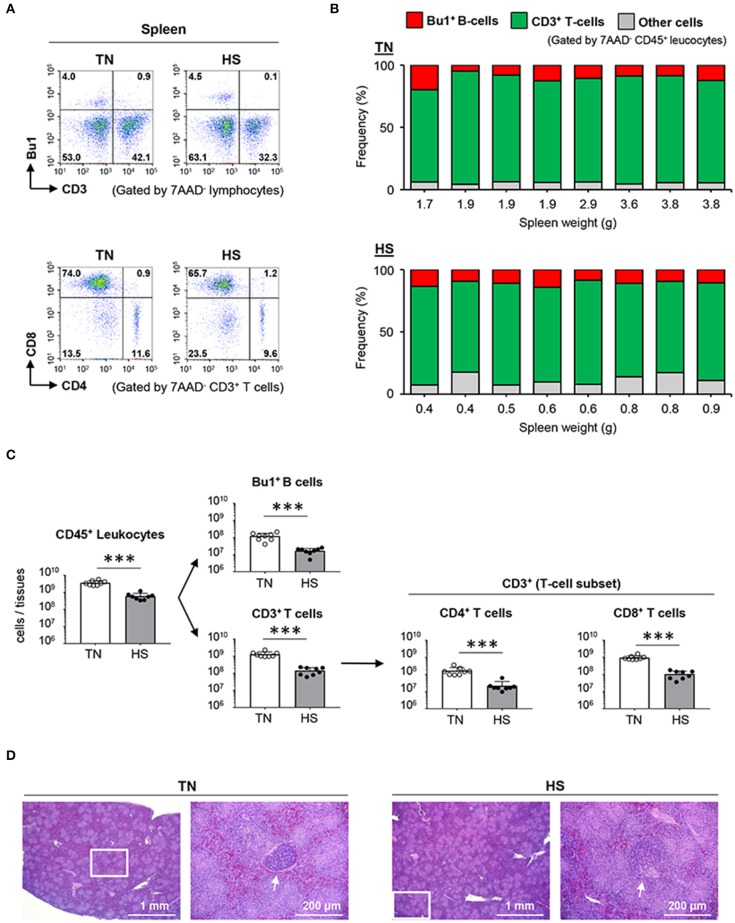
Effect of HS on lymphocytes and the spleen morphology of broiler chickens. Broiler chickens were maintained in the TN or HS condition for 14 days **(A,B)**. Representative flow cytometry profiles of 7AAD^−^CD45^+^ leucocytes and 7AAD^−^CD3^+^ T cells in the spleen at 36 days of age. **(C)** Cell components of splenic CD45^+^ leucocytes, including CD3^+^ T cells, Bu1^+^ B cells, and other cells, and absolute number of splenic CD45^+^ leucocytes, Bu1^+^ B cells, CD3^+^ T cells, CD4^+^ helper T cells, and CD8^+^ killer T cells were analyzed by flow cytometry **(D)**. Representative hematoxylin and eosin-stained histological sections of spleen at 36 days of age. Scale bar: 200 μm. Arrows: germinal center. The data are presented as means ± SE (*n* = 8/group). ***p < 0.001 compared with the TN group (unpaired two-tailed Student's *t-*test).

Given that humoral immune responses to produce antibodies are abolished by splenectomy in birds, resulting in poor development of lymphatic vessels and nodes (36), the spleen is believed to be crucial to the avian immune system. Thus, we hypothesized that the weak immune responses of broiler chickens to antigen immunized under the HS condition might be due to atrophy of the splenic structure. To elucidate the immunological status of the spleen under the HS condition, we evaluated the frequency and number of splenic lymphocytes by flow cytometry. There was no statistical difference observed in the frequency of splenic Bu1^+^ B cells and CD3^+^ T cells, including CD4^+^ T cells and CD8^+^ T cells, between the TN and HS groups (Figures 3A,B). However, importantly, the absolute number of Bu1^+^ B cells, CD3^+^ T cells, CD4^+^ T cells, and CD8^+^ T cells in the HS condition decreased significantly because of a reduction in the absolute number of total leukocytes expressing the pan-leukocyte marker CD45 (Figure 3C).

We next examined whether the depression of splenic B and T cells resulted from morphological abnormalities caused by HS. The basic structure of the avian spleen, which consists of red and white pulp, is equivalent to that of mammals; however, the absence of a marginal zone and the presence of two types of classified GCs based on the encapsulation of lymphocytes are unique phenotypes, only found in broiler chickens (37). Substantial differences in splenic red and white pulp were not observed between the HS and TN groups. However, the fully encapsulated GCs were rarely detected in the HS (not TN) group, whereas partially encapsulated GCs were equally found in both HS and TN groups (Figure 3D; Supplemental Material 1). These results indicate that the depression of lymphocytes and hypoplasia of the fully encapsulated GCs in the spleen could be an immediate cause of the insufficient humoral immune responses observed in heat-stressed broiler chickens.

### Immature B Cells in Bursal Follicles Decrease Severely in the HS Condition

**Figure 4:**
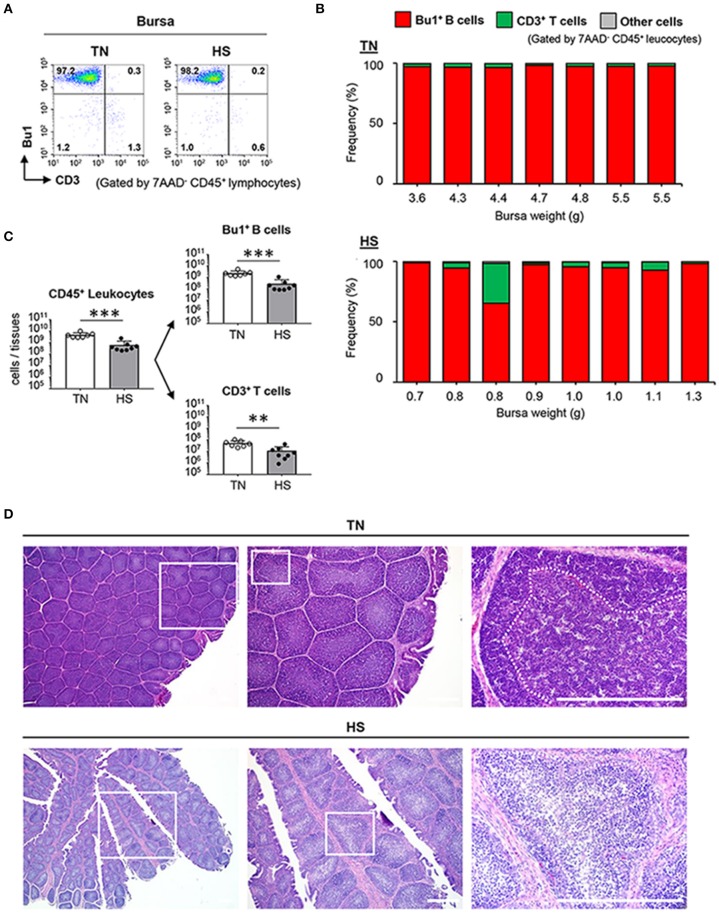
Effect of HS on the functional maturation of B cells in the bursa of Fabricius. Broiler chickens were maintained in either TN or HS condition for 14 days **(A,B)**. Representative flow cytometry profiles of 7AAD^−^CD45^+^ leucocytes in bursa of Fabricius at 36 days of age **(C)**. Cell components of bursal CD45^+^ leucocytes, including CD3^+^ T cells, Bu1^+^ B cells, other cells, and absolute number of bursal CD45^+^ leucocytes, Bu1^+^ B cells, and CD3^+^ T cells were analyzed by flow cytometry **(D)**. Representative hematoxylin and eosin-stained histological sections of bursa of Fabricius at 36 days of age. Scale bar: 200 μm. Dotted line: Border between cortex and medulla. The data are presented as mean ± SE (*n* = 7–8/group). **p < 0.01, ***p < 0.001 compared with the TN group (unpaired two-tailed Student's *t-*test). IFE, interfollicular epithelium; FAE, follicle-associated epithelium.

We next wondered whether the supply of mature B and T cells from primary lymphoid tissues to secondary lymphoid tissues was disturbed under the HS condition. Therefore, we analyzed the frequency and the absolute number of B cells in the bursa of Fabricius, which is responsible for both primary and secondary lymphoid tissues since the early stage of B cell development and subsequent Ig class-switching occurs partially in the tissue (38). Approximately 98% of bursal CD45^+^ leucocytes were identified as Bu1^+^ B cells and 1% of the remaining cells were identified as CD3^+^ T cells (Figures 4A,B). In contrast, the absolute number of Bu1^+^ B cells and CD3^+^ T cells decreased significantly in the HS group because of tissue atrophy with a low number of CD45^+^ leukocytes (Figure 4C). These results suggested that HS might influence the early stage of B cell development and differentiation in the bursa of Fabricius. Next, we histologically investigated the functional structure of the bursal folds, which consist of multiple follicles in which early stage B cells accumulate (39). HS did not affect development of the basic structure of the bursa of Fabricius, which is composed of multiple follicles and stromal tissues between follicles, both of which are covered by follicle-associated epithelium and interfollicular epithelium (Figure 4D). However, importantly, the cell density in the medulla and cortex of bursal follicles decreased severely under the HS (not TN) condition (Figure 4D). These results indicate that HS could influence the maintenance of the bursal structure, which plays a key role in the early stage of B cell development.

### Immature T Cells in the Thymic Cortex Are Sensitive to HS

**Figure 5:**
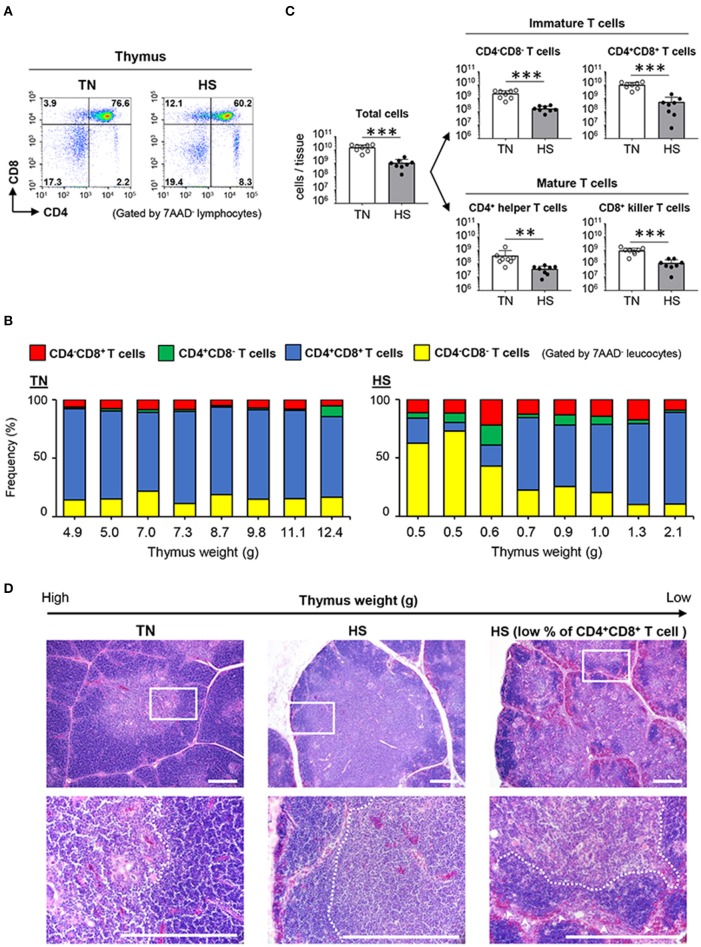
Effect of HS on the immature and mature T cells of the thymus. Broiler chickens were maintained in either TN or HS condition for 14 days **(A,B)**. Representative flow cytometry profiles of 7AAD^−^ leucocytes in the thymus at 36 days of age. **(C)** Cell components of thymic lymphocytes including CD4^−^CD8^−^ cells, CD4^+^CD8^+^ cells, CD4^+^CD8^−^ helper T cells, and CD4^−^CD8^+^ killer T cells, and absolute number of thymic T cell subsets were analyzed by flow cytometry. **(D)** Representative hematoxylin and eosin-stained histological sections of the thymus at 36 days of age. Scale bar: 200 μm. Dotted line: Border between cortex and medulla. Arrowheads: erythrocytes. The data are presented as means ± SE (*n* = 8/group). **p < 0.01, ***p < 0.001 compared with the TN group (unpaired two-tailed Student's *t-*test).

We next investigated the dynamics of T cells in the thymus, wherein positive and negative selections develop mature T cells that react to foreign (non-self) antigens. Under the TN condition, ~80% of thymocytes were identified as CD4^+^CD8^+^ T cells, which are precursor cells of either CD4^+^ helper or CD8^+^ killer T cells (Figures 5A,B). Notably, 5/8 of the chickens in the HS group showed a composition of thymocytes similar to that of the TN group; however, the other three chickens in the HS group, specifically those with the lowest thymus weights (0.5, 0.5, and 0.6 g), showed a significant decrease in the frequency of CD4^+^CD8^+^ T cells (Figure 5B). Since heat-stressed broiler chickens demonstrated the severe depletion of total thymocytes (Figure 5C), the number of each thymic T cell subset decreased severely under the HS condition (Figure 5C). These results suggest that HS might initially affect the maintenance of immature T cells and abolish the selection process during T cell development in the thymus.

To fully address whether HS affects the developmental processes of T cells in the thymic cortex and medulla, we compared the morphological structure of the thymus between the TN and HS groups. The location of each T cell subset in the thymus depends on its stage of maturity. The cortex is mainly involved in the development and proliferation of immature CD4^−^CD8^−^ T cells and CD4^+^CD8^+^ T cells, whereas the medulla is highly occupied by CD4^+^ helper T cells and CD8^+^ killer T cells (40). In the present study, the thymus of the heat-stressed broiler chickens displayed an unclear border between each lobe, and a thinner thymic cortex, when compared to those structures observed in the TN group (Figure 5D). Moreover, under the HS condition, massive thymic cortex degradation with erythrocyte influx was observed in the thymus wherein the frequency of CD4^+^CD8^+^ T cells decreased severely (Figure 5D). These findings indicated that HS could damage the thymic cortex, where immature T cells proliferate and differentiate into effector T cells. Together, our results demonstrate that atrophy of primary lymphoid tissues, with disruption of the early stage of lymphocyte differentiation and proliferation, could be the final phase of immune abnormality in broiler chickens bred under a high ambient temperature.

## Discussion

Recent studies have demonstrated that antigen-specific antibody responses do not increase as expected following immunization with commercialized vaccines in heat-stressed broiler chickens with an increased level of a stress mediator CORT (9). Moreover, HS tends to increase the susceptibility of broiler chickens to bacterial infections due to an increased proportion of pathogenic bacteria in the gut and an increased level of enteric permeability (13–16, 18, 41). Given the HS-induced immunological disorders, it is possible that global warming might be accompanied by a pandemic of infectious diseases, caused not only by pathogenic bacteria and viruses, but also by indigenous microorganisms (e.g., *Pseudomonas aeruginosa*) with weak pathogenicity. Therefore, the understanding of a series of immunological disorders in heat-stressed broiler chickens is of great importance to the poultry industry. Previous studies addressing abnormalities in immune functions of broiler chickens have mentioned that the weights of the thymus, bursa of Fabricius, and spleen decrease significantly under the HS condition (3, 8–10). In the lymphoid tissues, the function of mitochondria and the expression level of inflammatory cytokines (e.g., TNF-α, IFN-γ, IL-1, IL-2, IL-4, and IL-12) are affected by the HS (10, 30, 31). Further, the number of apoptosis cells and the level of apoptosis-related genes (e.g., p53, caspase 3, and caspase 9) increase in the lymphoid tissues under the HS condition (31). However, the functional and maturation status of immune cells present in atrophied primary and secondary lymphoid tissues had not yet been fully clarified. Therefore, according to our study results, we propose that the following findings are associated with the HS-induced immune abnormalities in broiler chickens: (i) severe reduction of the weight of immune tissues (i.e., thymus, bursa of Fabricius, and spleen) compared to those of non-lymphoid tissues; (ii) abnormal degradation of the thymic cortex, bursal lymphoid follicles, and splenic encapsulated GC; (iii) substantial lymphocyte depression in lymphoid tissues; and (iv) low titer of all antibody subclasses specific for BSA immunized as a prototype antigen.

To demonstrate immune function abnormalities in atrophied lymphoid tissues, we first examined the absolute number of T and B cells in the thymus, bursa of Fabricius, and spleen of heat-stressed broiler chickens. Although the frequency of CD3^+^ T cells and Bu1^+^ B cells in the CD45^+^ leukocyte population displayed no substantial differences between TN and HS conditions (Figures 3B, 4B), the absolute number of T and B cells in all three lymphoid tissues studied decreased significantly under the HS condition due their weight reduction with a decreased number of CD45^+^ leukocytes (Figures 3C, 4C, 5C). Another important finding obtained from our study was a change in the frequency of CD4^+^CD8^+^ T cells in the thymus. CD4^+^CD8^+^ T cells, which are the precursor of either CD4^+^ helper T cells or CD8^+^ T cells including cytotoxic T cells and γδ T cells, are the dominant T cell subset in the thymus in the healthy condition; however, in 3/8 of the chickens in the HS group, specifically those with the lowest thymus weights (0.5, 0.5, and 0.6 g), CD4^+^CD8^+^ T cells were not the dominant population (Figure 5B). The reason why the disruption of T and B cells occurs in the HS condition has not yet been fully elucidated; however, we have hypothesized that the heat-stressed broiler chickens might initiate metabolic reprogramming in an effort to adapt. To maintain tissue-specific homeostasis, sufficient amounts of nutrients (e.g., glucose, fatty acid, amino acids, minerals, and vitamins) need to be supplied to meet the specific tissue requirements. However, such nutritive substances might be insufficient because of reduced feed intake, which could be the most important sources under the HS condition. Regarding an importance of energy-yielding nutrients, to avoid overheating under the HS condition, chickens actively dissipate heat from their bodies by increasing evaporative cooling behavior (panting), which is an energy-intensive process (42). Under such circumstances, the limited amounts of energy-yielding substances are supplied to key metabolic tissues (e.g., muscle and liver). Additionally, such dysfunction of systemic energy homeostasis, induced by HS in broiler chickens, was accompanied by changes in plasma metabolites, including methionine metabolism-associated homocysteine, and cysteine (43). The methionine deficiency impairs the cell cycle, increasing apoptotic cells in the bursa of Fabricius (44). Furthermore, it has been known that zinc level is significantly reduced in the plasma, liver, and spleen of heat-stressed broiler chickens due to poor feed intake. Increased excretion of trace zinc results in reduced antioxidant actions against oxidative damage induced by HS (45, 46). Zinc deficiency also reduces proliferating cells in avian lymphoid tissues (47). Therefore, under the HS condition, the lymphoid tissues could receive insufficient macro-energy sources and/or micro-nutrients, resulting in the incomplete proliferation and differentiation of T and B cells.

In our immunization study, we demonstrated that the antigen-specific antibody titers of all subclasses (i.e., IgY, IgM, and IgA) were not elevated as expected under the HS (not TN) condition (Figure 2C). The amount of total IgY, which is the major Ig subclass in the plasma of broiler chickens, also did not increase following immunization (Figure 2B). These results might be due to HS-induced inflammation that inhibits acquired immune responses specific for the antigen immunized. Although inflammatory responses in heat-stressed broiler chickens were not examined in this study, past studies reported by other groups have demonstrated that inflammation occurred in the small intestine of heat-stressed broiler chickens with increased numbers of infiltrated heterophils and lymphocytes (3). The inflammatory cytokine (e.g., TNF-α and IL-6) levels are also elevated in the small intestine and plasma under the HS condition (18, 21, 40). Therefore, it is possible that in immune functions of heat-stressed broiler chickens they preferentially use the limited amount of energy substances for inflammatory (rather than humoral immune) responses. Another hypothesis to consider is the insufficient energy substances for B cell maturation, GC formation, and antibody production lead to immune abnormality in heat-stressed broiler chickens. Under the HS condition, insufficient amounts of glucose and glutamine may be transported into the antigen-activated B cells due to a decreasing amount of circulating glucose and glutamine, which account for 30–40% of amino acids in plasma (43, 48, 49). Given that glycolysis and glutamine oxidation are key metabolic pathways for antigen-activated B cells in peripheral tissues (50, 51), an unsatisfactory level of B cell maturation, GC formation, and antibody production might occur in the spleens of heat-stressed broiler chickens. An alternative proposal is the involvement of the stress-related hormone CORT. In humans and rabbits, increased CORT levels reduce the number of mature B cells and depress antibody production (52, 53). Moreover, intramuscularly administered CORT causes severe reduction of primary lymphoid tissues and suppresses lymphocyte proliferation in broiler chickens (54). Because we, and others, have demonstrated that HS significantly increased the level of plasma CORT (Figure 1C) (3, 8, 9, 13, 21, 30, 35), this could inhibit B cell proliferation and depress antibody production under the HS condition.

In summary, HS caused pathological atrophy of primary and secondary lymphoid tissues, reducing lymphocytes and degrading functional structure. Moreover, under the HS condition, the titer of antibodies that reacted specifically to the immunized antigen did not increase effectively, even after booster immunization. These findings led us to conclude that severe damage induced by HS in the primary and secondary lymphoid tissues induces the immune abnormalities in broiler chickens. Our findings provide a concrete rationale for excogitation of an appropriate approach to protect healthy chickens facing harsh environmental conditions, both at present and in the future.

## Data Availability Statement

The datasets generated for this study are available on request to the corresponding author.

## Ethics Statement

The animal study was reviewed and approved by Tohoku University Institutional Animal Care and Use Committee. Written informed consent was obtained from the owners for the participation of their animals in this study.

## Author Contributions

RH, SN, AM, MK, TN, and MT conceived and designed the study. RH, SN, KF, MK, and MT performed the experiments and analyzed the data. RH, TN, and MT wrote the paper. All authors read and approved the final manuscript.

### Conflict of Interest

The authors declare that the research was conducted in the absence of any commercial or financial relationships that could be construed as a potential conflict of interest.
