# Supplementary figures and images for: Heat Stress Causes Immune Abnormalities via Massive Damage to Effect Proliferation and Differentiation of Lymphocytes in Broiler Chickens
Source: Front Vet Sci. 2020 Feb 7;7:46. doi: 10.3389/fvets.2020.00046 (PMC7020782; doi:10.3389/fvets.2020.00046)

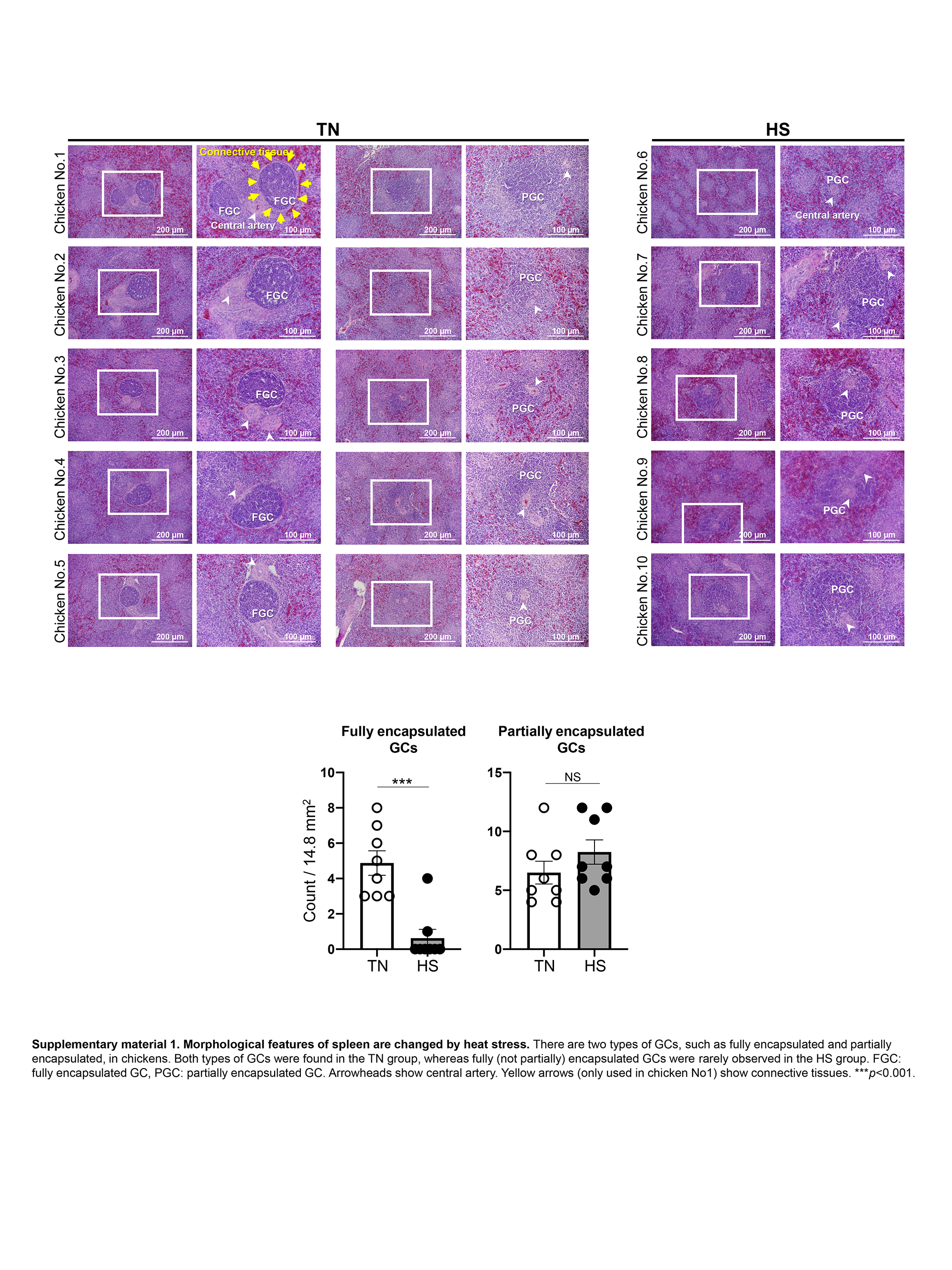

Supplement: Supplementary file 1 [file Image_1.TIF]
